# Evaluation of a Zn–2Ag–1.8Au–0.2V Alloy for Absorbable Biocompatible Materials

**DOI:** 10.3390/ma13010056

**Published:** 2019-12-20

**Authors:** Ping Li, Christine Schille, Ernst Schweizer, Evi Kimmerle-Müller, Frank Rupp, Xingting Han, Alexander Heiss, Andreas Richter, Claudia Legner, Ulrich E. Klotz, Jürgen Geis-Gerstorfer, Lutz Scheideler

**Affiliations:** 1 Section Medical Materials Science and Technology, University Hospital Tübingen, Osianderstrasse 2–8, 72076 Tübingen, Germany; Christine.Schille@med.uni-tuebingen.de (C.S.); Ernst.Schweizer@med.uni-tuebingen.de (E.S.); Evi.kimmerle-mueller@med.uni-tuebingen.de (E.K.-M.); Frank.Rupp@med.uni-tuebingen.de (F.R.); xingting.han@student.uni-tuebingen.de (X.H.); Juergen.Geis-Gerstorfer@med.uni-tuebingen.de (J.G.-G.); Lutz.Scheideler@med.uni-tuebingen.de (L.S.); 2 Research Institute for Precious Metals and Metals Chemistry (fem), Katharinenstrasse 17, 73525 Schwäbisch Gmünd, Germany; heiss@fem-online.de (A.H.); richter@fem-online.de (A.R.); legner@fem-online.de (C.L.); klotz@fem-online.de (U.E.K.)

**Keywords:** biocompatible materials, biocompatibility, bioactivity, zinc, absorbable, biofilm

## Abstract

Zinc (Zn) and Zn-based alloys have been proposed as a new generation of absorbable metals mainly owing to the moderate degradation behavior of zinc between magnesium and iron. Nonetheless, mechanical strength of pure Zn is relatively poor, making it insufficient for the majority of clinical applications. In this study, a novel Zn–2Ag–1.8Au–0.2V (wt.%) alloy (Zn–Ag–Au–V) was fabricated and investigated for use as a potential absorbable biocompatible material. Microstructural characterization indicated an effective grain-refining effect on the Zn alloy after a thermomechanical treatment. Compared to pure Zn, the Zn–Ag–Au–V alloy showed significantly enhanced mechanical properties, with a yield strength of 168 MPa, an ultimate tensile strength of 233 MPa, and an elongation of 17%. Immersion test indicated that the degradation rate of the Zn–Ag–Au–V alloy in Dulbecco’s phosphate buffered saline was approximately 7.34 ± 0.64 μm/year, thus being slightly lower than that of pure Zn. Biocompatibility tests with L929 and Saos-2 cells showed a moderate cytotoxicity, alloy extracts at 16.7%, and 10% concentration did not affect metabolic activity and cell proliferation. Plaque formation in vitro was reduced, the Zn–Ag–Au–V surface inhibited adhesion and biofilm formation by the early oral colonizer *Streptococcus gordonii*, indicating antibacterial properties of the alloy.

## 1. Introduction

Absorbable (biodegradable) metals refer to metals degrading safely within the body. In that field, magnesium (Mg), iron (Fe), zinc (Zn), and their alloys have gained increasing interest as absorbable biocompatible materials potentially useable for vascular stents and osteosynthesis implants [1,2,3,4,5]. An appropriate degradation behavior is critical to maintain its mechanical integrity as well as the bioactivity of the implant during the whole tissue remodeling period. Regarding Mg and its alloys, the main drawback is the too-fast degradation process accompanied by the accumulation of hydrogen in the tissue, making Mg-based materials not only too insufficient to provide mechanical integrity but impeding tissue healing as well [6,7]. In contrast, Fe and its alloys degrade too slowly in vivo, leading to long-term retention in the body [8,9].

Zn-based alloys have been considered and proposed as a new generation of absorbable metals mainly owing to their moderate corrosion behavior, the standard corrosion potential of Zn (−0.76 V_SCE_) being between Fe (−0.44 V_SCE_) and Mg (−2.37 V_SCE_) [10,11]. More importantly, elemental Zn is an essential element for biological functions. The average daily zinc intake is 4–14 mg/day for humans [12]. Notably, a previous in vivo study demonstrated for Zn wires implanted into the murine artery a steady degradation behavior and no local toxicity over 20 months after implantation [13]. Moreover, Li et al. [14] reported around Zn–1X (Mg, Ca, and Sr) pins implanted into mouse femora new bone formation and observed remodeling. Nonetheless, the strength of pure Zn is relatively poor, insufficient for the majority of clinical applications. For instance, for current osteosynthesis materials, an ultimate tensile strength (UTS) of approximately 300 MPa is claimed [4]. In contrast, as cast pure Zn has a UTS of approximately 10 MPa, which is far lower than the strength requirements for clinical applications [2,3,4,15].

To date, Zn-based alloys with superior strength have been achieved by adding different alloying elements, with or without a thermomechanical treatment. Alloying elements such as Mg, Cu, Ag, Ca, Sr, Al, Mn, Li, Fe, and rare eath elements, etc. have been fabricated and investigated, mainly including the Zn–Mg alloy system, Zn–Cu alloy system, and Zn–Ag alloy system [4,16,17,18]. The selection of alloying elements should be based not only on the improvement of mechanical properties but also on the consideration of biocompatibility and bioactivity. In previous studies, adding elemental Ag to Zn alloys has been demonstrated to enhance the mechanical properties efficiently [18,19,20,21,22]. Also, the additional trait of adding elemental Ag is its antibacterial property [18,23]. Moreover, for clinical use, radiopacity is a significant requirement of medical devices such as endovascular stents and catheters, beneficial for the less invasive surgery and follow-up observation [10]. Gold (Au) has been used in biomaterials and dental materials because of its flexibility and radiopacity. Ohyama et al. [24] tested Au stents for the treatment of intracranial aneurysms in vivo. The results indicated that the Au stents possessed superior radiopacity and biocompatibility in the experimental endovascular procedures. In addition, trace vanadium (V) as an alloying element can further refine microstructure and improve age hardening of Mg–Zn alloys [25]. Therefore, based on the above considerations, Ag, Au, and V can bring advantages as the alloying elements for Zn-based alloys.

In this study, a novel Zn–2Ag–1.8Au–0.2V (wt.%) quaternary alloy (denoted as Zn–Ag–Au–V) was fabricated and evaluated as a potential absorbable biomaterial. A series of thermomechanical treatments were used to refine the microstructure. The microstructure and mechanical properties of Zn–Ag–Au–V were investigated. In addition, the in vitro degradation behavior, cytotoxicity, and antibacterial properties were further evaluated.

## 2. Materials and Methods

### 2.1. Materials Preparation

Phase diagrams were calculated using the Thermo-Calc software package (TCS) and the associated TCS Nobel metal-based alloy database (TCNOBL1). The alloy was prepared analogously to the previous study [18]. In short, the elements were melted under 1 bar argon in a graphite crucible and then cast in a rectangular graphite mold. Homogenization in a furnace at 300 °C was followed by hot rolling at 200 °C and annealing at 390 °C for 15 min. Finally, plates with a dimension 30 mm × 10 mm × 0.5 mm for immersion test and of 7 mm × 7 mm × 0.5 mm for cytotoxicity and antibacterial evaluation were cut, respectively.

Samples were mechanically ground with carbide paper up to grit 600 (CarbiMet P1200, Buehler, Düsseldorf, Germany) using a grinding machine (Metaserv, Buehler, Düsseldorf, Germany). Afterwards, samples were ultrasonically cleaned with absolute ethanol for 10 min (Sonorex K102H, Bandelin, Berlin, Germany). Samples were further disinfected with ultraviolet radiation for at least 1 h in a workbench.

### 2.2. Microstructure Characterization and Mechanical Properties Test

Metallographic specimens were cut, ground, polished, and then etched with 2% Nital, a mixture of HNO_3_ and EtOH. The microstructure of samples was observed by optical microscopy (OM, Zeiss Axioplan 2; Carl Zeiss, Oberkochen, Germany). Scanning electron microscopy (SEM, Zeiss Auriga 60, Carl Zeiss Microscopy GmbH, Oberkochen, Germany) was performed to determine the phase morphology. Additionally, phase analysis was performed on bulk samples by X-ray diffraction (XRD) using a diffractometer D8 Discover (Bruker AXS GmbH, Karlsruhe, Germany) in GADDS-configuration (“General Area Detector Diffraction System”). The Cu Kα X-ray beam was limited by a collimator and a snout with 1-mm diameter to hit the sample. A Vantec-500 area detector by Bruker AXS placed in 25-cm distance to the sample was used to collect the detector frames with an integration time of 300 s per frame. The 2D diffraction patterns were integrated along the tilt-angle and merged in the software package Diffrac.EVA 5.1 by Bruker AXS resulting in the one-dimensional diffraction pattern for phase analysis. Phase analysis was done by comparing measured reflections to the ICDD-PDF 2 database (International Centre for Diffraction Data – Powder Diffraction File).

According to ISO 10002–1: 2001 [26], the tensile properties including 0.2% yield strength (YS_0.2_), ultimate tensile strength, and elongation were evaluated. The specimens with 3 mm in diameter were carried out using a universal testing machine (Zwick Z100HT, Zwick GmbH, Ulm, Germany). Tensile tests were performed at room temperature with a testing speed of 1.5 mm min^−1^ and then increased to a strain-controlled strain rate of 2.5 × 10^−3^ s^−1^. In addition, the Vickers hardness (diamond pyramid hardness, HV1) of samples was measured at room temperature on metallographically polished cross-sections, and the applied load was 1 kg.

### 2.3. Immersion Test

A semi-static immersion test was performed in Dulbecco’s phosphate buffered saline (DPBS, Life Technologies, Paisley, UK) at 37 ± 1 °C for 49 days, according to ISO 10271: 2011 [27]. The composition of DPBS is listed in Table 1 and compared to the human extracellular fluid [28,29]. The Zn–Ag–Au–V alloys were immersed in DPBS with a ratio of surface area to solution volume of 1.0 cm^2^/mL, and pure Zn as a control. The DPBS was refreshed at certain time points (1, 4, 7, 14, 21, 28, 35, and 42 days). Metallic ion release in DPBS was detected using an inductively coupled plasma optical emission spectrometer (ICP-OES, Optima 4300 DV, Perkin Elmer, Rodgau, Germany), and the pH values were measured. Prior to the ICP-OES measurement, tiny degradation particles in the solution were dissolved by pure HNO_3_, as described previously [30,31]. After immersion, degradation products were removed by glycine (NH_2_CH_2_COOH) (250 g/L) for 10 min, according to ISO 8407: 2009 [32]. Afterwards, the degradation rate was calculated by weight loss using the following equation according to the American Society for Testing and Materials standard (ASTM G31-12a) [33]:

Degradation rate (μm/year) = 8.76 × 10^7^ × (ΔW) / (D × A × T). (1)

Here, ΔW is weight loss. D (g/cm^3^) is the sample density. A (cm^2^) is the surface area. T (h) is the immersion time. In addition, the surface morphology and chemical composition before and after removing degradation products were evaluated by a scanning electron microscope (SEM) equipped with an energy dispersive X-ray (EDX) analyzer at 10 kV (LEO 1430, Carl Zeiss GmbH, Oberkochen, Germany).

### 2.4. Cytotoxicity Test

An extract test was performed to evaluate cytotoxicity according to ISO 10993-5: 2009 [34]. Mouse fibroblast cells (L929, DSMZ GmbH, Braunschweig, Germany) and human osteosarcoma cells (Saos-2, DSMZ GmbH, Braunschweig, Germany) were used. L929 fibroblasts were cultured in Dulbecco’s modified Eagle medium (DMEM, Life Technologies, Paisley, UK) supplemented with 10% heat-inactivated fetal bovine serum (FBS, Life Technologies, Paisley, UK). Saos-2 osteoblasts were cultured in McCoy’s 5A (Sigma-Aldrich Chemie GmbH, Steinheim, Germany) supplemented with 15% FBS. Both cell culture media were supplemented with 1% penicillin/streptomycin (Life Technologies, Grand Island, NY, USA) and 1% GlutaMAX (Life Technologies, Paisley, UK). Both cells were grown in 75 cm^2^ flasks (Costar, Corning, Tewksbury, MA, USA) under standard cell culture conditions.

Tested samples were immersed in the respective cell culture media with FBS under cell culture conditions for 24 h, according to ISO 10993-12: 2012 [35]. Ti–6Al–4V alloy was used as a negative control, and pure Cu as a positive control. The ratio of surface area to extraction medium was 3.0 cm^2^/mL. After incubation for 24 h, the original extracts were diluted with the respective fresh cell culture media for 3, 6, and 10 times (labeled as 33.3%, 16.7%, and 10%, respectively), according to the recommendation in [36]. The metallic ion concentrations in the extracts were detected by ICP-OES, and the pH values were determined.

Live/dead staining assay was performed to evaluate cell morphology and cell viability exposed to sample extracts. L929 and Saos-2 cells were seeded in 12-well plates (2.4 mL per well) at a density of 3 × 10^4^ cells/cm^2^ and then incubated overnight. Afterwards, cell culture media were replaced by the respective sample extracts. After 24 h, cell morphologies were photographed using an inverted microscope (CK2, Olympus, Tokyo, Japan) equipped with a remote control digital single-lens reflex (DSLR 550D, Canon, Tokyo, Japan). Subsequently, sample extracts were removed and cells were gently rinsed with Hank’s balanced salt solution (HBSS, Boichrom AG, Berlin, Germany). Live/dead working solution was prepared consisting of 25 μg/mL fluorescein diacetate (FDA) and 1.25 μg/mL ethidium bromide (EB) in 10 mL HBSS. Afterwards, 1.5 mL working solution was added to each well, followed by incubation for 10 min in darkness. After gentle rinsing with HBSS, cells were observed using a fluorescence microscope (Optiphot-2, Nikon, Tokyo, Japan).

The inhibition of cell metabolic activity and cell proliferation of L929 and Saos-2 cells exposed to different sample extracts was quantitatively assessed. Two assays were used: A tetrazolium assay (XTT, Roche, Mannheim, Germany) and a bromodeoxyuridine assay (BrdU, Roche, Mannheim, Germany), respectively. Briefly, cells were seeded in 96-well plates at a density of 3 × 10^4^ cells/cm^2^ (200 μL per well) and incubated overnight. Afterwards, the media were replaced with 150 μL of the respective different diluted extracts. After 24 h incubation, 50 μL XTT working solution was added to each well for 2 h. Then, the absorbance was measured using a microplate ELISA reader with Gen5 Software (Eon Reader, Biotek Instruments GmbH, Bad Friedrichshall, Germany) at the wavelength of 450/620 nm. Additionally, to determine cell proliferation in the logarithmic growth phase between 24 h and 48 h after seeding, 15 μL BrdU labeling reagent was added to each well 24 h after cell seeding. After 24 h incubation, proliferative activities of L929 and Saos-2 were determined by BrdU incorporation, following manufacturer’s instructions. The absorbance was measured at 450/690 nm.

### 2.5. Antibacterial Test

To evaluate antibacterial properties, the Zn–Ag–Au–V alloy and Ti reference samples were inoculated with *Streptococcus gordonii* strain DL1 (*S. gordonii*), and evaluated using a fluorescence-based live/dead staining (Bacterial Viability Kit L13152, Invitrogen, Carlsbad, CA, USA). *S. gordonii* were grown as stationary suspension culture in Schaedler medium (Beckton Dickinson GmbH, Heidelberg, Germany) and incubated overnight at 37 °C. Afterwards, 4 mL *S. gordonii* suspension was added to each sample in 6-well plates and cultivated at 37 °C for 12 h and 24 h, respectively. After incubation for 12 h and 24 h, samples were rinsed two times with HBSS. *S. gordonii* adhesion on the surfaces was evaluated by live/dead staining assay, following manufacturer’s instructions. The initial biofilm formation and bacterial adhesion on the samples were observed using a fluorescence microscope.

### 2.6. Statistical Analysis

Statistical analyses were conducted using SPSS 22.0 software (IBM Corporation, Chicago, IL, USA). All quantitative data were given as the mean ± standard deviation. Statistically significant differences between two groups were analyzed by a two-tailed, unpaired Student’s t-test, and the significance level was set to *p* < 0.05.

## 3. Results and Discussion

### 3.1. Microstructure and Mechanical Properties

**Figure 1:**
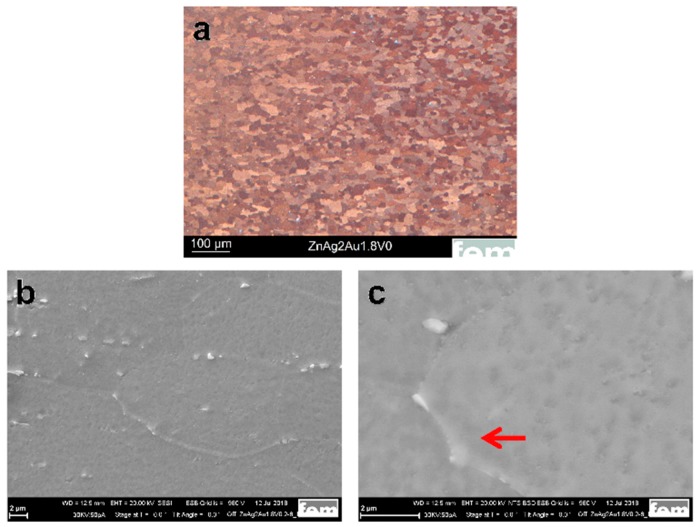
Microstructure characterization of the Zn–Ag–Au–V alloy after thermomechanical treatment and additional precipitation hardening. (**a**) Optical micrograph (OM) of the Zn–Ag–Au–V alloy, (**b**) scanning electron microscope (SEM) investigation of the specimen at an accelerating voltage of 20 kV, and (**c**) backscatter electron imaging (BSE) at 20 kV. The red arrow indicates the presence of coarser precipitates along the grain boundaries while finer precipitates formed within the grains.

Figure 1a shows the optical image of the Zn–Ag–Au–V specimen after thermomechanical treatment and additional precipitation hardening. As shown in Figure 1b, the second phase particles were randomly distributed along the Zn grain boundaries. Moreover, a micrograph acquired by backscatter electron imaging (BSE) at 20 kV is shown in Figure 1c. The BSE imaging revealed the presence of second phase precipitates dispersed in the interior and at the boundaries of the Zn grains, similar to Zn–4Ag [18].

In our study, the average grain size of the Zn–Ag–Au–V alloy can be significantly refined via the process optimization of the casting. Specifically, a longer holding time of the melt causes better mixing (homogeneity). By adding a vibration during the solidification of the cast rod, the dendritic peaks in the cast rod broke, resulting in the formation of more numerous and finer grains. Furthermore, as-cast pure Zn and its alloys exhibited dendritic structure with anisotropy, leading to relatively low strength [5]. For the Zn–Ag–Au–V alloy, the dendritic cast structure was successfully transformed into a globular structure through forming and heat treatment (solution annealing). Fine precipitates were formed after thermomechanical treatment (solution annealing and precipitation), as shown in Figure 1b.

**Figure 2:**
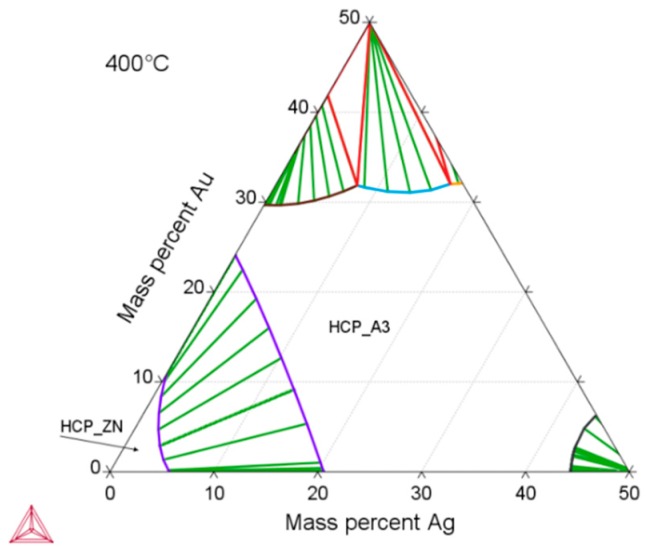
Zn-rich part of the calculated ternary Zn–Ag–Au phase diagram using the Thermo-Calc software and TCNOBL1. The depicted isothermal section at 400 °C is characterized by a Zn solid solution (HCP_ZN) and a stability area of solid solution series of ε-phase (HCP_A3).

**Figure 3:**
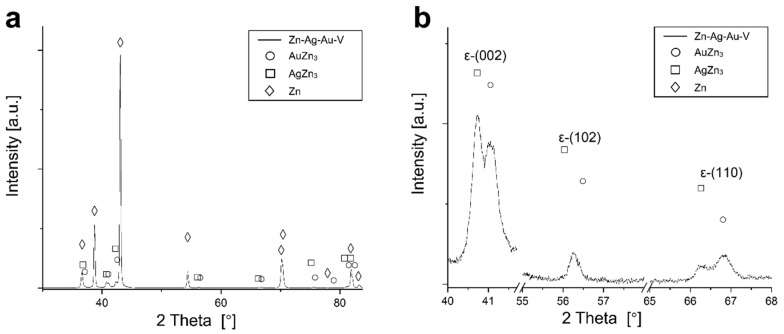
XRD pattern of the Zn–Ag–Au–V alloy after thermomechanical treatment. (**a**) Diffraction pattern of the Zn–Ag–Au–V alloy; (**b**) detail view of the reflections of the precipitations of ε-phases (002), (102), and (110).

According to thermodynamic calculations, Ag and Au showed a temperature-dependent solubility in the Zn matrix (Zn solid solution), i.e., the HCP_ZN area (hexagonal closest packing HCP) shrinks on cooling. Consequently, another phase HCP_A3 was expected to form representing the ε-phase, as depicted in Figure 2. The evaluation of the diffraction patterns confirmed that besides metallic zinc (ICDD-PDF #87-0713) as the main phase, a second phase corresponding to the ε-phase could be detected (Figure 3a) in low concentration. The precipitated ε-phase occurred in the binary systems zinc–silver and zinc–gold as AgZn_3_ (ICDD-PDF #25-1325), respectively, AuZn_3_ (ICDD-PDF #65-8027). The diffraction pattern shows two solid solution members of the ε-phase with distinct differences in their fitted lattice parameters (Figure 3b). Surprisingly, the (102)-reflection of the ε-phase showed no separation; instead, the measured single reflection was positioned in the middle between the two expected reflections of the silver- and gold-rich ε-phases. We suppose that the coexistence of two ε-phase members was caused by local chemical inhomogeneities in the sample, i.e., solid solution crystallites with variable or graded chemical composition.

The mechanical properties of the Zn–Ag–Au–V alloy are shown in Table 2. The strength of Zn–Ag–Au–V alloy was significantly enhanced compared to pure Zn [4,5]. After thermomechanical treatment and additional precipitation hardening, respectively, the mechanical properties were assessed. A comparison revealed that the 0.2% yield strength (YS_0.2_), ultimate tensile strength (UTS), and hardness (HV1) increased slightly while elongation decreased significantly. Obviously, the UTS value of the Zn–Ag–Au–V alloy was higher than the polymeric materials and close to the Mg-based alloy (WE43), as listed in Table 2. Despite the remarkable reduction in elongation of the Zn–Ag–Au–V alloy induced by precipitation hardening, it still met the minimum requirements for stents and orthopedic internal fixation implants of approximately 15% according to [2].

### 3.2. In Vitro Degradation Behavior

**Figure 4:**
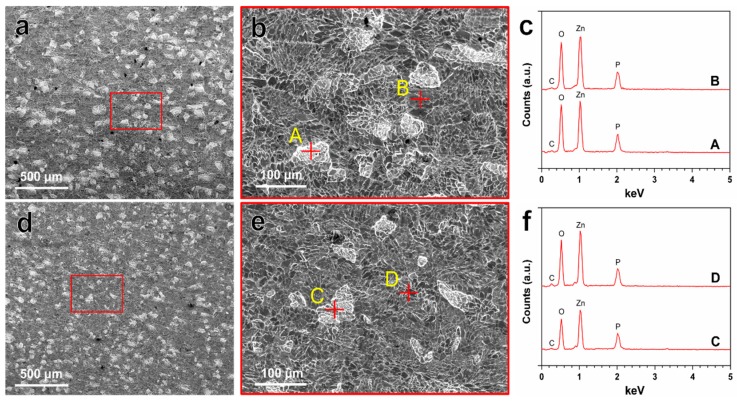
Representative SEM images of the degradation products on the surfaces of pure Zn (**a**,**b**) and Zn–Ag–Au–V alloy (**d**,**e**) after immersion test before removing degradation products. EDX results (**c**,**f**) representative points marked with yellow letters A–B and C–D are given in (**b**,**e**), respectively.

The in vitro degradation behavior of Zn–Ag–Au–V alloy was evaluated by immersion test in DPBS for 49 days. The degradation rate of Zn–Ag–Au–V alloy in DPBS solution was 7.34 ± 0.64 μm/year, which is slightly lower than that of pure Zn, 8.66 ± 0.35 μm/year. Figure 4 indicates the surface morphologies and chemical composition of degradation products detected by SEM-EDX after immersion for 49 days. Inhomogeneous degradation layers were formed covering the surfaces, as shown in Figure 4a,d. Obviously, “crystal-like” degradation products were observed at high magnification (Figure 4b,e), in line with previous studies [19,37]. Moreover, EDX analysis (Figure 4c,f) showed that these degradation products were mainly composed of Zn, P, O, and C, indicating that these degradation layers might be phosphates and carbonates, as previously reported [38,39,40].

**Figure 5:**
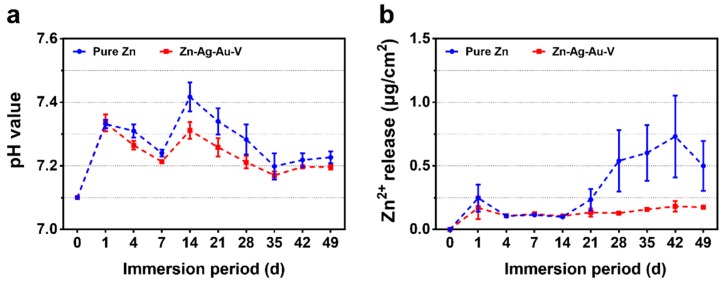
Degradation kinetics of pure Zn and Zn–Ag–Au–V alloy in Dulbecco’s phosphate buffered saline (DPBS) for 49 days of immersion: (**a**) pH value and (**b**) Zn ion release.

Figure 5 illustrates the degradation kinetics of pure Zn and Zn–Ag–Au–V alloy in DPBS during 49 days of immersion. All samples maintained pH values in the range from 7.15 to 7.45 mainly due to the buffering effect in the DPBS. In addition, the cumulative Zn^2+^ release of Zn–Ag–Au–V alloy was lower than that of pure Zn. In the initial period, the Zn^2+^ release of Zn–Ag–Au–V alloy showed no significant difference compared to pure Zn. However, the Zn^2+^ release of the pure Zn from the 28th day on was lower than that of the Zn–Ag–Au–V alloy. The Ag, Au, and V ions released in DPBS were below the detection limit of the instrument (50 μg/L).

**Figure 6:**
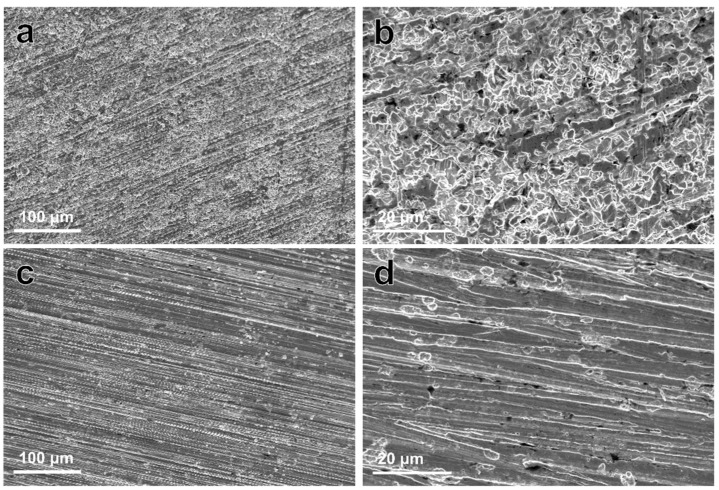
Representative low- and high-magnification SEM images of pure Zn (**a**,**b**), and Zn–Ag–Au–V alloy (**c**,**d**) after removing degradation products (low-magnification, 500×; and high-magnification, 3000×).

Figure 6 shows the corroded morphologies of samples after removing the degradation products. The corrosion morphology of the Zn–Ag–Au–V alloy appeared relatively uniform, without large pits or extensive localized corrosion, similar to that of pure Zn. It can be inferred that there was little micro-galvanic corrosion of the Zn–Ag–Au–V alloy.

Based on our results, the in vitro degradation behavior of the Zn–Ag–Au–V alloy in DPBS can be deduced. When samples were immersed in DPBS, initial degradation reactions involved the following series of anodic dissolution of the metal and the cathodic reduction of oxygen, referring to Equations (2) and (3) [2,38,41]. Due to the dissolution of Zn, released OH^−^ increased the pH value in the DPBS. As shown in Figure 5, ICP-OES result shows that Zn^2+^ was quickly released and increased the pH value in the initial period. With the Zn^2+^ and OH^−^ released, precipitations of Zn(OH)_2_ and ZnO were formed on the surfaces, referring to Equations (4) and (5) [38,42]. Due to Zn(OH)_2_ and ZnO layers formed, the release of Zn^2+^ and the pH value trend became stable. Similar results of initial degradation products on the surface could be observed in vitro as well as in vivo [41,42,43]. In addition, HPO_4_^2−^ in the DPBS can react with released Zn^2+^ to form insoluble zinc phosphate (Equation (6)) [4]. The insoluble zinc phosphate thermodynamically represented a more stable passive film compared to Zn(OH)_2_ and ZnO layers [3,4]. The EDX analysis of the biodegradation revealed that the products were mainly composed of Zn, P, and O, most likely reflecting the presence of zinc oxide, zinc hydroxide, and zinc phosphate. The corrosion layers on the surface tended to be porous and show cracks, considered as potential transport paths for corrosion, leading to chloride diffusing through the layer and attacking the Zn-matrix [44]. It was found that the surface was covered by deeper corroded areas or degradation holes after removing degradation products, as shown in Figure 6.

Anodic reaction:

2Zn (s) → 2Zn^2+^ (aq) + 4e^−^ (2)

Cathodic reaction:

2H_2_O + O_2_ + 4e^−^ → 4OH^−^ (3)

Zn(OH)_2_ formation:

Zn^2+^ + 2OH^−^ → Zn(OH)_2_ (4)

ZnO formation:

Zn^2+^ + 2OH^−^ → ZnO + H_2_O Zn(OH)_2_ → ZnO + H_2_O (5)

Zinc phosphate formation:

3Zn^2+^ + 2OH^−^ + 2H_2_O + 2HPO_4_^2−^ → Zn_3_(PO_4_)_2_·4H_2_O (6)

In our results, the Zn–Ag–Au–V alloy showed a relatively low degradation rate of 7.34 ± 0.64 μm/year, which was far lower than reported for most Zn-based alloys [2,45]. One explanation might be the high concentration of HPO_4_^2-^ in the DPBS (4.2 mM), as listed in Table 1. The degradation rate could be significantly decreased due to the formation of insoluble zinc phosphate in this medium. Unlike Zn(OH)_2_, the compact Zn phosphates as passive film could protect the Zn substrate and delay the degradation process [4]. Furthermore, compared to pure Zn, the degradation behavior of Zn–Ag–Au–V alloys can be mainly influenced by the alloying elements, especially size/distribution of the metal phases. Sikora-Jasinska et al. [19] demonstrated that the degradation rates of Zn–Ag alloys are higher than that of pure Zn. They proposed that the Zn–5Ag and Zn–7Ag are subjected to micro-galvanic corrosion mainly due to a distinct galvanic coupling between ε-AgZn_3_ dendrites and Zn matrix. As shown in Figure 6, the corroded morphology of Zn–Ag–Au–V was relatively uniform, probably attributed to grain refinement and homogenization of chemical distribution after the thermomechanical treatment [4].

### 3.3. Cytotoxicity Evaluation

Table 3 shows the mean Zn ion concentration and pH values in Zn–Ag–Au–V alloy extracts. The mean Zn^2+^ concentration in undiluted extracts of DMEM + 10% FBS and McCoy’s 5A + 15% FBS were 738.9 μM and 313.7 μM, respectively. The other metallic ion concentrations (Ag^2+^, Au^2+^, V^2+^) in the extracts were below the detection limit of the ICP-OES (<50 μg/L), indicating extremely low free ions in the extracts. Probably these ions were bound in degradation particles [46]. Moreover, no apparent increase in pH value (Δ pH < 0.5) was observed due to the buffer effect of the media (Table 1).

**Figure 7:**
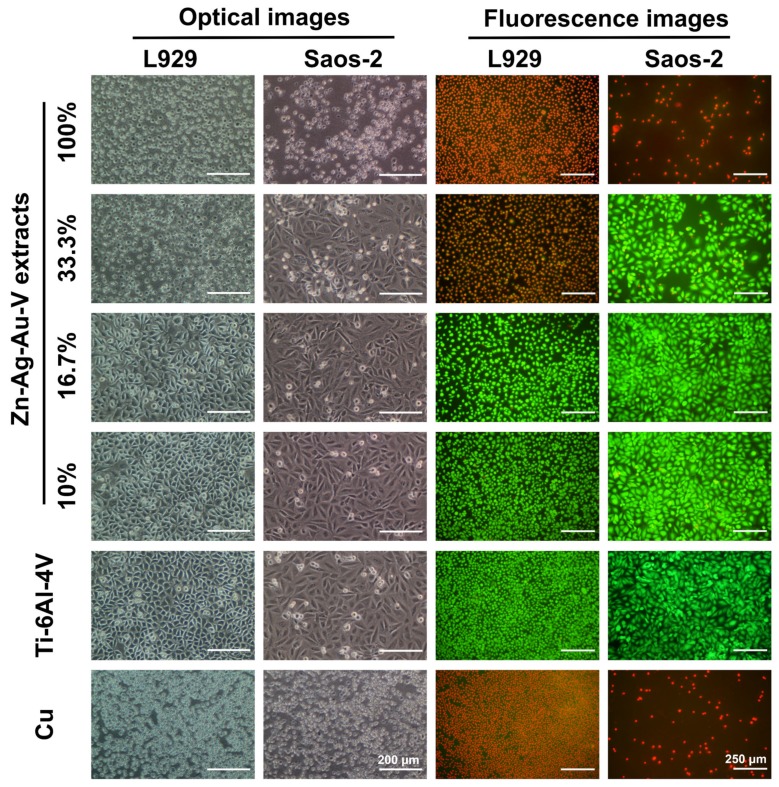
Optical images and fluorescence images of L929 cells and Saos-2 cells cultured in different extracts of Zn–Ag–Au–V alloy after incubation for 24 h. Ti–6Al–4V alloy was used as a negative control and pure Cu as a positive control. Green fluorescence indicates viable cells stained with fluorescein diacetate (FDA), and red fluorescence indicates apoptotic cells with compromised membrane integrity, stained with ethidium bromide (EB).

As shown in Figure 7, cell viability and morphology were observed by optical microscope and live/dead staining assay. In the 100% extracts, almost all L929 and Saos-2 cells showed round-shaped morphologies and red staining, in line with those of the positive control (pure Cu). In contrast, L929 and Saos-2 cells cultured in the 16.7% and 10% extracts established a well-pronounced attachment, while only a few round-shaped cells were floating in the extracts. Accordingly, fluorescent images revealed that almost all cells were alive (green staining) and only a quite limited number of dead cells (red staining) could be randomly observed in the 16.7% and 10% extracts, respectively. Notably, L929 and Saos-2 cells exposed to 33% extracts showed an inconsistent result. While Saos-2 cells were predominantly spindle-shaped and viable (green), L929 mainly showed round-shaped morphologies and were apoptotic (red).

**Figure 8:**
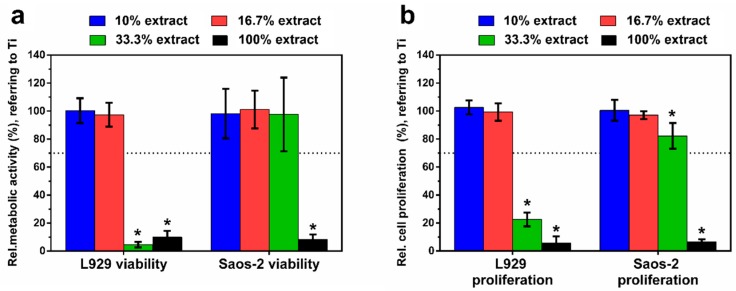
Relative metabolic activity (**a**) and cell proliferation (**b**) of L929 and Saos-2 cells cultured in different extracts of Zn–Ag–Au–V alloy after incubation for 24 h. Relative metabolic activity and cell proliferation were normalized to Ti–6Al–4V as a negative control. Undiluted extracts (100% extracts) display significantly lower cell viability and proliferation analyzed by Student’s t-test; * represents *p* < 0.05 when compared to the negative control. The means of two independent experiment are shown with respective standard deviations. Dashed line presents the cut-off level between nontoxic and toxic effects according to ISO 10993-5.

Figure 8a depicts the relative metabolic activity of L929 and Saos-2 cells, respectively, exposed to the different extracts of Zn–Ag–Au–V alloy for 24 h. The highly concentrated 100% extract decreased metabolic activity and proliferation of L929 and Saos-2 cells significantly, compared to the negative control (*p* < 0.05). This was in perfect agreement with the previous microscopic results regarding the toxic effects according to ISO 10993-5 [34]. Relative metabolic activities of L929 and Saos-2 in the lower concentrated 16.7% and 10% extracts were always close to the control value, showing no statistically significant difference compared to the negative control (*p* > 0.05). Interestingly, the metabolic activity of Saos-2 in 33% extract was considerably higher than that of L929, indicating a lower sensitivity of the Saos-2 cell at medium extract concentration. Figure 8b shows cell proliferation of L929 and Saos-2 cells, respectively, exposed to the different extracts of Zn–Ag–Au–V alloy for 24 h and determined by BrdU assay. The results of the proliferation test corresponded to the metabolic activity data, confirming almost total inhibition for the undiluted original extracts, and no significant inhibition for the lowest extract concentration (10% and 16.7%).

Regarding absorbable biocompatible materials, biocompatibility is of primary importance. Previous reports underline the excellent biocompatibility of Zn-based alloys when implanted into vessels (intravascular) or bone (endosseous) in animal studies [13,14,42]. However, Zn and its alloys exhibited apparent cytotoxic effects observed in in vitro tests [17,29,47]. It can be stated that there was an obvious discrepancy between the in vitro and in vivo results concerning biocompatibility of Zn-based alloys, probably due to the different degradation environments in vivo: Tissue, cells, blood flow, etc.

In fact, a standardized extract test assesses the influence of degradation products released from material on the cellular reaction via artificial corrosion media. Our results showed that undiluted extracts of Zn–Ag–Au–V alloy led to almost total inhibition of cell viability and proliferation, indicating an obvious toxic effect. Analysis of sample extracts revealed that the concentrations of the metallic ions Ag^2+^, Au^2+^, V^2+^ were extremely low, under the detection limit. Thus, cytotoxicity was mainly attributed to the Zn^2+^ concentration in the extracts, as previously reported [18,29,30]. In this study, the mean Zn^2+^ concentration in 100% extracts of DMEM + 10% FBS and McCoy’s 5A + 15% FBS were 738.9 μM and 313.7 μM, respectively, which was far beyond most cellular tolerance limits, such as, e.g., for L929 fibroblasts (<80 μM) [30], U-2 OS osteoblast (<120 μM) [30], vascular smooth muscle cells (<80 μM) [48], and primary human coronary artery endothelial cells (<100 μM) [49]. As expected, the cytotoxic effect was decreased after dilution of sample extracts, consistent with previous studies [18,50]. Notably, the released Zn ion concentration in DMEM + 10% FBS (738.9 μM) was almost two-fold higher than the that in McCoy’s 5A + 15% FBS (313.7 μM), indicating a different initial degradation process of the Zn–Ag–Au–V alloy in both cell culture media. One factor might be the putative inhibition of the degradation process in McCoy’s 5A due to the high concentration of HPO_4_^2−^ (Table 1).

Current ISO standards (10993-5 and -12) have only limited value for the evaluation of absorbable metals since they were developed mainly for nondegradable, bioinert materials. A previous study demonstrated that the standards were not able to mimic the physiological metabolism, especially clearance of the degradation products after implantation via dissolution in interstitial fluid, the lymphatic system, or the blood circulation in vivo. A minimal 6-fold dilution to a maximal 10-fold dilution of extracts were recommended for screening of the cytotoxic potential of Mg-based alloys [36]. Likewise, this recommendation might be suitable for the in vitro cytotoxicity test of Zn-based alloys. At higher extract dilutions, the Zn–Ag–Au–V alloy exhibited acceptable toxicity towards L929 and Saos-2 cells. Nevertheless, further in vivo biocompatibility tests are required.

### 3.4. Antibacterial Evaluation

**Figure 9:**
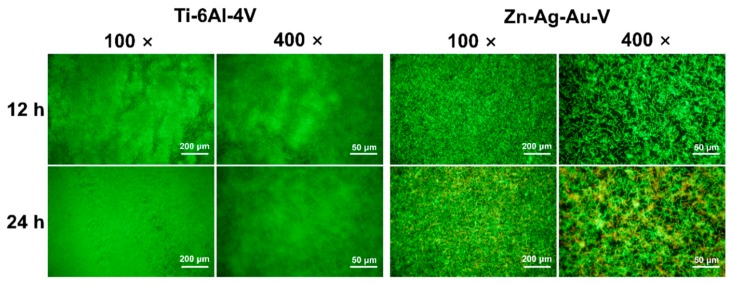
Representative low- and high-magnification fluorescent images of biofilm formation on Ti–6Al–4V and Zn–Ag–Au–V alloy surfaces with *Streptococcus gordonii* after 12 h and 24 h determined by live/dead staining assay (low-magnification, 100×; and high-magnification, 400×).

As shown in Figure 9, the antibacterial properties of the Zn–Ag–Au–V alloy were evaluated by live/dead staining. After 12 h incubation with *S. gordonii*, a pronounced, bright green fluorescence was observed on the Ti–6Al–4V surface. In contrast, a relatively weak green fluorescence on the Zn–Ag–Au–V surface was observed. After incubation for 24 h, a dense layer of viable *S. gordonii* on the surface of Ti–6Al–4V samples was observed, indicating biofilm formation. In contrast, a relatively thin green fluorescence showed that the *S. gordonii* chains consisted of less viable and nonviable bacteria on the Zn–Ag–Au–V surfaces, indicating that initial *S. gordonii* colonization and biofilm formation were inhibited.

Recently, Zn-based alloys have been demonstrated to be promising materials for craniomaxillofacial osteosynthesis implants [5,51]. An intraoral approach is the main access for maxillofacial surgery, and probably increases the risk of infection caused by oral bacteria. Potentially pathogenic microorganisms related to transoral maxillofacial surgery mainly include streptococci, anaerobic Gram-negative rods as well as anaerobic Gram-positive cocci [52]. *S. gordonii* has been widely recognized as a pioneer oral strain in the process of early bacterial colonization, which plays a key role in forming a pathogenic plaque [53]. Thereby, *S. gordonii* was selected in this study as a model bacterium to analyze initial oral biofilm formation. In principle, ideal biomaterials should not only have excellent biocompatibility but ideally possess antibacterial properties as well. To date, the antibacterial properties of the Zn-based alloys, such as Zn–4Ag alloy, Zn–Cu alloys and Zn–Al–Mg alloys, etc., have been reported [18,54,55,56]. In our study, the Zn–Ag–Au–V alloy displayed antibacterial effects. Nonetheless, the antibacterial mechanism of the Zn-based alloys was still not understood, probably attributable to the degradation products, such as Zn^2+^, Ag^2+^, and OH^−^ release, etc. According to the Pourbaix diagrams of Zn in an acidic environment (i.e., *S. gordonii* growth medium after 24 h seeding, pH value: Approximately from 4.3 to 4.5), pure Zn has no tendency to be passivated and mainly releases free Zn ion, referring to Equation (3). Herein, the potential mechanism of the bacterial adhesion inhibited by Zn ions might be caused by generating reactive oxygen species or inhibiting multiple activities of bacteria [57,58]. Moreover, other surface-confined reactions might play a role. Ag ion, as one alloying element, possesses effective antibacterial properties as well. As previously reported, Ag ions may induce bacterial stress that can lead to the viable but a nonculturable state, which depends on the released Ag ion rate [59,60]. In a previous study, an increased Ag content (from 2% to 6%) enhanced the antibacterial properties of Mg–Ag alloys [61]. In addition, the cathodic reaction was accompanied by a local increase in pH (OH^−^ release). An in vitro antibacterial test of Mg alloy demonstrated that an alkaline shift in pH was a critical factor in determining bacterial viability [62]. Thus, with the synergy of these above factors, the Zn–Ag–Au–V alloy in vitro test possessed the potential to inhibit *S. gordonii* adhesion and colonization.

Within the limitations of this study, the Zn–Ag–Au–V alloy exhibited promising performance. Mechanical properties of the Zn–Ag–Au–V alloy were apparently improved after thermomechanical treatment. Nevertheless, there is still a need to further improve mechanical strength, especially for load-bearing applications. Concerning the in vitro degradation evaluation, the DPBS solution was among the most frequently used simulated body fluids. However, DPBS lacked the organic components of human extracellular fluids, such as cells, proteins, amino acids, etc. Thus, the degradation behavior of the Zn–Ag–Au–V alloy in the different simulated body fluids should be further explored. Furthermore, current standardized cytotoxicity tests have been controversially discussed to predict the biocompatibility of degradable materials like Zn and Zn alloys. It is necessary to perform additional in vivo tests to evaluate the biocompatibility of the Zn–Ag–Au–V alloy. The antibacterial properties of the Zn–Ag–Au–V alloy were demonstrated in this study by qualitative assessment via live/dead fluorescent labelling as well as by crystal violet staining (data not shown). However, the antibacterial effect of the surface should be further evaluated via sensitive quantitative tests, e.g., a luminescence assay.

## 4. Conclusions

In this study, we fabricated and investigated a new Zn–Ag–Au–V alloy as a potential material for absorbable biocompatible implants. The mechanical tests demonstrated markedly improved properties. The yield strength, ultimate tensile strength, and elongation of the Zn–Ag–Au–V alloy were 168 MPa, 233 MPa and 17%, respectively, after thermomechanical treatment. Furthermore, the degradation rate of the Zn–Ag–Au–V alloy in DPBS was 7.34 ± 0.64 μm/year, which is slightly lower than that of pure Zn, which is 8.66 ± 0.35 μm/year. Severe localized corrosion of the Zn–Ag–Au–V alloy was also not observed. Regarding the cytotoxicity evaluation, the Zn–Ag–Au–V alloy showed acceptable toxicity in the results obtained with cells exposed to 10% and 16.7% extracts. However, notable toxic effects in undiluted extracts were observed. On the other hand, the in vitro antibacterial test demonstrated that the Zn–Ag–Au–V alloy had the potential for inhibition of surface colonization by oral bacteria. Initial *S. gordonii* adhesion and colonization was markedly decreased compared to the reference, Ti–6Al–4V alloy. In conclusion, the investigated Zn–Ag–Au–V alloy indicated excellent strength, uniform degradation behavior, acceptable cytotoxicity, and effective antibacterial properties in vitro, rendering it a promising biodegradable material.

## Figures and Tables

**Table 1:** Main composition of the employed solutions compared with the human extracellular fluid [28,29].

Composition	Human Extracellular Fluid		DPBS	DMEM	McCoy’s 5A
	Blood Plasma	Interstitial Fluid			
Inorganic Ions (mM)					
Na^+^	142.0	139.0	146.0	127.3	141.0
K^+^	4.2	4.0	4.1	5.3	5.4
Mg^2+^	0.8	0.7	-	0.8	0.8
Ca^2+^	1.3	1.2	-	1.8	1.2
Cl^−^	106.0	108.0	140.6	90.8	117.2
SO_4_^2−^	0.5	0.5	-	0.8	0.8
HPO_4_^2−^	2.0	2.0	9.5	0.9	4.2
HCO_3_^−^	24.0	28.3	-	44.1	26.2
Organic Components					
Protein	1.2 (mM)	0.2 (mM)	-	-	-
Glucose (mM)	5.6	5.6	-	4.5	16.6
Amino acids	2.0 (mM)	2.0 (mM)	-	1.6 (g/L)	0.4 (g/L)
Concentrations of Buffering Agents (mM)					
HCO_3_^−^	24.0	28.3	-	44.1	26.2
HPO_4_^2−^	2.0	2.0	9.5	0.9	4.2
Tris-HCl	-	-	-	25.0	-
HPr	16.0–18.0	-	-	-	-
Total	42.0–44.0	30.3	9.5	70.0	30.4

**Table 2:** Mechanical properties of the Zn–Ag–Au–V alloy.

Materials	Processing	Mechanical Properties				Reference
		YS_0.2_ (MPa)	UTS (MPa)	Elongation (%)	Hardness (HV1)	
Zn–Ag–Au–V	Thermomechanical treatment	129	231	59	61	In this study
Zn–Ag–Au–V	Thermomechanical treatment *	168	233	17	96	In this study
Pure Zn	As cast	10	18	0.3	38	[2]
Pure Zn	As extruded	35	60	3.5	-	[2]
Pure Zn	As hot rolled	30–110	50–140	5.8–36	39	[2]
PLA	-	-	48–53	30–240	-	[5]
PGA	-	-	60–99	1.5–20	-	[5]
WE43	As extruded	195.2	280.6	10.3	-	[5]

* Additional precipitation hardening.

**Table 3:** Mean Zn ion concentration and pH value of Zn–Ag–Au–V alloy extracts.

Extracts	Zn ion Concentration (μM)		pH Value	
	DMEM + 10% FBS	McCoy’s 5A + 15% FBS	DMEM + 10% FBS	McCoy’s 5A + 15% FBS
100%	738.9	313.7	8.0	8.2
33.3%	249.3	111.9	7.9	8.2
16.7%	126.5	61.3	7.8	8.1
10%	77.2	41.0	7.8	8.1
Original	4.3	6.0	7.6	8.1

## References

[1] Neacsu P.N., Ion R., Mitran V.I., Staras A., Cîmpean A. (2014). State of the art and recent patents on Mg-based biodegradable bone implants. Recent Pat. Regen. Med..

[2] Katarivas Levy G., Goldman J., Aghion E. (2017). The prospects of zinc as a structural material for biodegradable implants—A review paper. Metals.

[3] Mostaed E., Sikora-Jasinska M., Drelich J.W., Vedani M. (2018). Zinc-based alloys for degradable vascular stent applications. Acta Biomater..

[4] Venezuela J., Dargusch M. (2019). The influence of alloying and fabrication techniques on the mechanical properties, biodegradability and biocompatibility of zinc: A comprehensive review. Acta Biomater..

[5] Li P., Zhang W., Dai J., Xepapadeas A.B., Schweizer E., Alexander D., Scheideler L., Zhou C., Zhang H., Wan G. (2019). Investigation of zinc-copper alloys as potential materials for craniomaxillofacial osteosynthesis implants. Mater. Sci. Eng. C.

[6] Geis-Gerstorfer J., Schille C., Schweizer E., Rupp F., Scheideler L., Reichel H.-P., Hort N., Nolte A., Wendel H.-P. (2011). Blood triggered corrosion of magnesium alloys. Mater. Sci. Eng. B.

[7] Neacsu P., Staras A., Voicu S., Ionascu I., Soare T., Uzun S., Cojocaru V., Pandele A., Croitoru S., Miculescu F. (2017). Characterization and in vitro and in vivo assessment of a novel cellulose acetate-coated Mg-based alloy for orthopedic applications. Materials.

[8] Pierson D., Edick J., Tauscher A., Pokorney E., Bowen P., Gelbaugh J., Stinson J., Getty H., Lee C.H., Drelich J. (2012). A simplified in vivo approach for evaluating the bioabsorbable behavior of candidate stent materials. J. Biomed. Mater. Res. A.

[9] Li Y., Jahr H., Lietaert K., Pavanram P., Yilmaz A., Fockaert L., Leeflang M., Pouran B., Gonzalez-Garcia Y., Weinans H. (2018). Additively manufactured biodegradable porous iron. Acta Biomater..

[10] Zheng Y., Xu X., Xu Z., Wang J.-Q., Cai H. (2017). Metallic Biomaterials: New Directions and Technologies.

[11] Eliaz N. (2019). Corrosion of metallic biomaterials: A review. Materials.

[12] Su Y., Cockerill I., Wang Y., Qin Y.-X., Chang L., Zheng Y., Zhu D. (2018). Zinc-based biomaterials for regeneration and therapy. Trends Biotechnol..

[13] Drelich A., Zhao S., Guillory R.J., Drelich J.W., Goldman J. (2017). Long-term surveillance of zinc implant in murine artery: Surprisingly steady biocorrosion rate. Acta Biomater..

[14] Li H., Xie X., Zheng Y., Cong Y., Zhou F., Qiu K., Wang X., Chen S., Huang L., Tian L. (2015). Development of biodegradable Zn-1x binary alloys with nutrient alloying elements Mg, Ca and Sr. Sci. Rep..

[15] Li Y., Pavanram P., Zhou J., Lietaert K., Taheri P., Li W., San H., Leeflang M., Mol J., Jahr H. (2019). Additively manufactured biodegradable porous zinc. Acta Biomater..

[16] Kubásek J., Dvorský D., Šedý J., Msallamová Š., Levorová J., Foltán R., Vojtěch D. (2019). The fundamental comparison of Zn–2Mg and Mg–4Y–3Re alloys as a perspective biodegradable materials. Materials.

[17] Dambatta M., Murni N., Izman S., Kurniawan D., Froemming G., Hermawan H. (2015). In vitro degradation and cell viability assessment of Zn–3Mg alloy for biodegradable bone implants. Proc. Inst. Mech. Eng. J.

[18] Li P., Schille C., Schweizer E., Rupp F., Heiss A., Legner C., Klotz U.E., Geis-Gerstorfer J., Scheideler L. (2018). Mechanical characteristics, in vitro degradation, cytotoxicity, and antibacterial evaluation of Zn-4.0 Ag alloy as a biodegradable material. Int. J. Mol. Sci..

[19] Sikora-Jasinska M., Mostaed E., Mostaed A., Beanland R., Mantovani D., Vedani M. (2017). Fabrication, mechanical properties and in vitro degradation behavior of newly developed Zn Ag alloys for degradable implant applications. Mater. Sci. Eng. C.

[20] Wątroba M., Bednarczyk W., Kawałko J., Mech K., Marciszko M., Boelter G., Banzhaf M., Bała P. (2019). Design of novel Zn–Ag–Zr alloy with enhanced strength as a potential biodegradable implant material. Mater. Des..

[21] Hehrlein C., Schorch B., Kress N., Arab A., von zur Mühlen C., Bode C., Epting T., Haberstroh J., Mey L., Schwarzbach H. (2019). Zn-alloy provides a novel platform for mechanically stable bioresorbable vascular stents. PLoS ONE.

[22] Shuai C., Xue L., Gao C., Yang Y., Peng S., Zhang Y. (2018). Selective laser melting of Zn–Ag alloys for bone repair: Microstructure, mechanical properties and degradation behaviour. Virtual Phys. Prototyp..

[23] Sotoudehbagha P., Sheibani S., Khakbiz M., Ebrahimi-Barough S., Hermawan H. (2018). Novel antibacterial biodegradable Fe–Mn–Ag alloys produced by mechanical alloying. Mater. Sci. Eng. C.

[24] Ohyama T., Nishide T., Iwata H., Taki W. (2004). Development of gold stents for the treatment of intracranial aneurysms: An experimental study in a canine model. AJNR Am. J. Neuroradiol..

[25] Buha J. (2008). Grain refinement and improved age hardening of Mg–Zn alloy by a trace amount of V. Acta Mater..

[26] International Organization for Standardization (2001). Tensile Testing of Metallic Materials. Method of Test at Ambient Temperature.

[27] International Organization for Standardization (2011). Dentistry-Corrosion Test Methods for Metallic Materials.

[28] Schille C., Braun M., Wendel H., Scheideler L., Hort N., Reichel H.-P., Schweizer E., Geis-Gerstorfer J. (2011). Corrosion of experimental magnesium alloys in blood and PBS: A gravimetric and microscopic evaluation. Mater. Sci. Eng. B.

[29] Li P., Schille C., Schweizer E., Kimmerle-Müller E., Rupp F., Heiss A., Legner C., Klotz U.E., Geis-Gerstorfer J., Scheideler L. (2019). Selection of extraction medium influences cytotoxicity of zinc and its alloys. Acta Biomater..

[30] Kubásek J., Vojtěch D., Jablonská E., Pospíšilová I., Lipov J., Ruml T. (2016). Structure, mechanical characteristics and in vitro degradation, cytotoxicity, genotoxicity and mutagenicity of novel biodegradable Zn–Mg alloys. Mater. Sci. Eng. C.

[31] Jablonská E., Vojtěch D., Fousová M., Kubásek J., Lipov J., Fojt J., Ruml T. (2016). Influence of surface pre-treatment on the cytocompatibility of a novel biodegradable ZnMg alloy. Mater. Sci. Eng. C.

[32] International Organization for Standardization (2009). Corrosion of Metals and Alloys–Removal of Corrosion Products from Corrosion Test Specimens.

[33] American Society for Testing and Materials (2017). Standard Guide for Laboratory Immersion Corrosion Testing of Metals.

[34] International Organization for Standardization (2009). Biological Evaluation of Medical Devices–Part 5: Tests for In Vitro Cytotoxicity.

[35] International Organization for Standardization (2012). Biological Evaluation of Medical Devices–Part 12: Sample Preparation and Reference Materials.

[36] Wang J., Witte F., Xi T., Zheng Y., Yang K., Yang Y., Zhao D., Meng J., Li Y., Li W. (2015). Recommendation for modifying current cytotoxicity testing standards for biodegradable magnesium-based materials. Acta Biomater..

[37] Liu X., Sun J., Yang Y., Pu Z., Zheng Y. (2015). In vitro investigation of ultra-pure Zn and its mini-tube as potential bioabsorbable stent material. Mater. Lett..

[38] Chen Y., Zhang W., Maitz M.F., Chen M., Zhang H., Mao J., Zhao Y., Huang N., Wan G. (2016). Comparative corrosion behavior of Zn with Fe and Mg in the course of immersion degradation in phosphate buffered saline. Corros. Sci..

[39] Paramitha D., Chabaud S., Bolduc S., Hermawan H. (2019). Biological assessment of Zn–based absorbable metals for ureteral stent applications. Materials.

[40] Zhang Y., Yan Y., Xu X., Lu Y., Chen L., Li D., Dai Y., Kang Y., Yu K. (2019). Investigation on the microstructure, mechanical properties, in vitro degradation behavior and biocompatibility of newly developed Zn–0.8% Li–(Mg, Ag) alloys for guided bone regeneration. Mater. Sci. Eng. C.

[41] Bowen P.K., Drelich J., Goldman J. (2013). Zinc exhibits ideal physiological corrosion behavior for bioabsorbable stents. Adv. Mater..

[42] Yang H., Wang C., Liu C., Chen H., Wu Y., Han J., Jia Z., Lin W., Zhang D., Li W. (2017). Evolution of the degradation mechanism of pure zinc stent in the one-year study of rabbit abdominal aorta model. Biomaterials.

[43] Liu X., Sun J., Qiu K., Yang Y., Pu Z., Li L., Zheng Y. (2016). Effects of alloying elements (Ca and Sr) on microstructure, mechanical property and in vitro corrosion behavior of biodegradable Zn–1.5 Mg alloy. J. Alloy Comp..

[44] Liu L., Meng Y., Dong C., Yan Y., Volinsky A.A., Wang L.-N. (2018). Initial formation of corrosion products on pure zinc in simulated body fluid. J. Mater. Sci. Technol..

[45] Champagne S., Mostaed E., Safizadeh F., Ghali E., Vedani M., Hermawan H. (2019). In vitro degradation of absorbable zinc alloys in artificial urine. Materials.

[46] Li P., Dai J., Schweizer E., Rupp F., Heiss A., Richter A., Klotz U.E., Geis-Gerstorfer J., Scheideler L., Alexander D. (2020). Response of human periosteal cells to degradation products of zinc and its alloy. Mater. Sci. Eng. C.

[47] Murni N., Dambatta M., Yeap S., Froemming G.R.A., Hermawan H. (2015). Cytotoxicity evaluation of biodegradable Zn–3Mg alloy toward normal human osteoblast cells. Mater. Sci. Eng. C.

[48] Ma J., Zhao N., Zhu D. (2016). Bioabsorbable zinc ion induced biphasic cellular responses in vascular smooth muscle cells. Sci. Rep..

[49] Ma J., Zhao N., Zhu D. (2015). Endothelial cellular responses to biodegradable metal zinc. ACS Biomater. Sci. Eng..

[50] Wang C., Yang H., Li X., Zheng Y. (2016). In vitro evaluation of the feasibility of commercial Zn alloys as biodegradable metals. J. Mater. Sci. Technol..

[51] Wang X., Shao X., Dai T., Xu F., Zhou J.G., Qu G., Tian L., Liu B., Liu Y. (2019). In vivo study of the efficacy, biosafety, and degradation of a zinc alloy osteosynthesis system. Acta Biomater..

[52] Spaey Y.J., Bettens R.M., Mommaerts M.Y., Adriaens J., Van Landuyt H.W., Abeloos J.V., De Clercq C.A., Lamoral P.R., Neyt L.F. (2005). A prospective study on infectious complications in orthognathic surgery. J. Cranio Maxillofac. Surg..

[53] Loo C., Corliss D., Ganeshkumar N. (2000). Streptococcus gordonii biofilm formation: Identification of genes that code for biofilm phenotypes. J. Bacteriol..

[54] Tang Z., Niu J., Huang H., Zhang H., Pei J., Ou J., Yuan G. (2017). Potential biodegradable Zn-Cu binary alloys developed for cardiovascular implant applications. J. Mech. Behav. Biomed. Mater..

[55] Niu J., Tang Z., Huang H., Pei J., Zhang H., Yuan G., Ding W. (2016). Research on a Zn-Cu alloy as a biodegradable material for potential vascular stents application. Mater. Sci. Eng. C.

[56] Bakhsheshi-Rad H., Hamzah E., Low H., Kasiri-Asgarani M., Farahany S., Akbari E., Cho M. (2017). Fabrication of biodegradable Zn-Al-Mg alloy: Mechanical properties, corrosion behavior, cytotoxicity and antibacterial activities. Mater. Sci. Eng. C.

[57] Hu H., Zhang W., Qiao Y., Jiang X., Liu X., Ding C. (2012). Antibacterial activity and increased bone marrow stem cell functions of Zn-incorporated TiO_2_ coatings on titanium. Acta Biomater..

[58] Phan T.N., Buckner T., Sheng J., Baldeck J., Marquis R. (2004). Physiologic actions of zinc related to inhibition of acid and alkali production by oral streptococci in suspensions and biofilms. Mol. Oral Microbiol..

[59] Heiss A., Freisinger B., Held-Föhn E. (2017). Enhanced antibacterial activity of silver-ruthenium coated hollow microparticles. Biointerphases.

[60] Königs A.M., Flemming H.C., Wingender J. (2015). Nanosilver induces a non-culturable but metabolically active state in Pseudomonas aeruginosa. Front. Microbiol..

[61] Tie D., Feyerabend F., Mueller W.-D., Schade R., Liefeith K., Kainer K.U., Willumeit R. (2012). Antibacterial biodegradable Mg-Ag alloys. Eur. Cell Mater..

[62] Brooks E.K., Ahn R., Tobias M.E., Hansen L.A., Luke-Marshall N.R., Wild L., Campagnari A.A., Ehrensberger M.T. (2018). Magnesium alloy AZ91 exhibits antimicrobial properties in vitro but not *in vivo*. J. Biomed. Mater. Res. B.

